# Waxed Cloth Cones

**Published:** 1848-07

**Authors:** 


### "WAXED CLOTH CONES."

We received, a short time since, of our colleague, Dr. Brown, of St. Louis, a couple of very ingeniously contrived Waxed Cloth Cones, for arresting Alveolar Hæmorrhage; they appear to be made of strips of muslin coated with wax, then rolled so as to very much resemble roots of teeth—of these various sizes might be kept, so that when a case of hæmorrhage occurs, we have only to select one as near the size of the socket as possible, and after removing the blood from the same, force one of these cones in, and thus by compression arrest the hæmorrhage.

They are certainly exceedingly convenient, and we think possees one or two advantages over any thing else for the purpose we have seen—for instance, if warmed by the heat of the mouth or otherwise, they will assume the form of the cavity, and close it up as perfectly as possible. CIN. ED.

